# Supplementary material for: Common and distinct transcriptional signatures of mammalian embryonic lethality
Source: Nat Commun. 2019 Jun 26;10:2792. doi: 10.1038/s41467-019-10642-x (PMC6594971; doi:10.1038/s41467-019-10642-x)
Supplement: Supplementary file 1 — Supplementary information [file 41467_2019_10642_MOESM1_ESM.pdf]

Common and distinct transcriptional signatures of mammalian embryonic lethality

Collins et al.

Supplementary Figures

Supplementary Figure 1. Comparison of RNA-seq on dissected tissues and whole embryos.

Supplementary Figure 2. Example of DESeq2 delay analysis.

Supplementary Figure 3. No Delay genes.

Supplementary Figure 4. Analysis of repeat deregulation in *Dhx35* and *Morc2a* mutant lines.

Supplementary Figure 5. Intron retention in *Dhx35*.

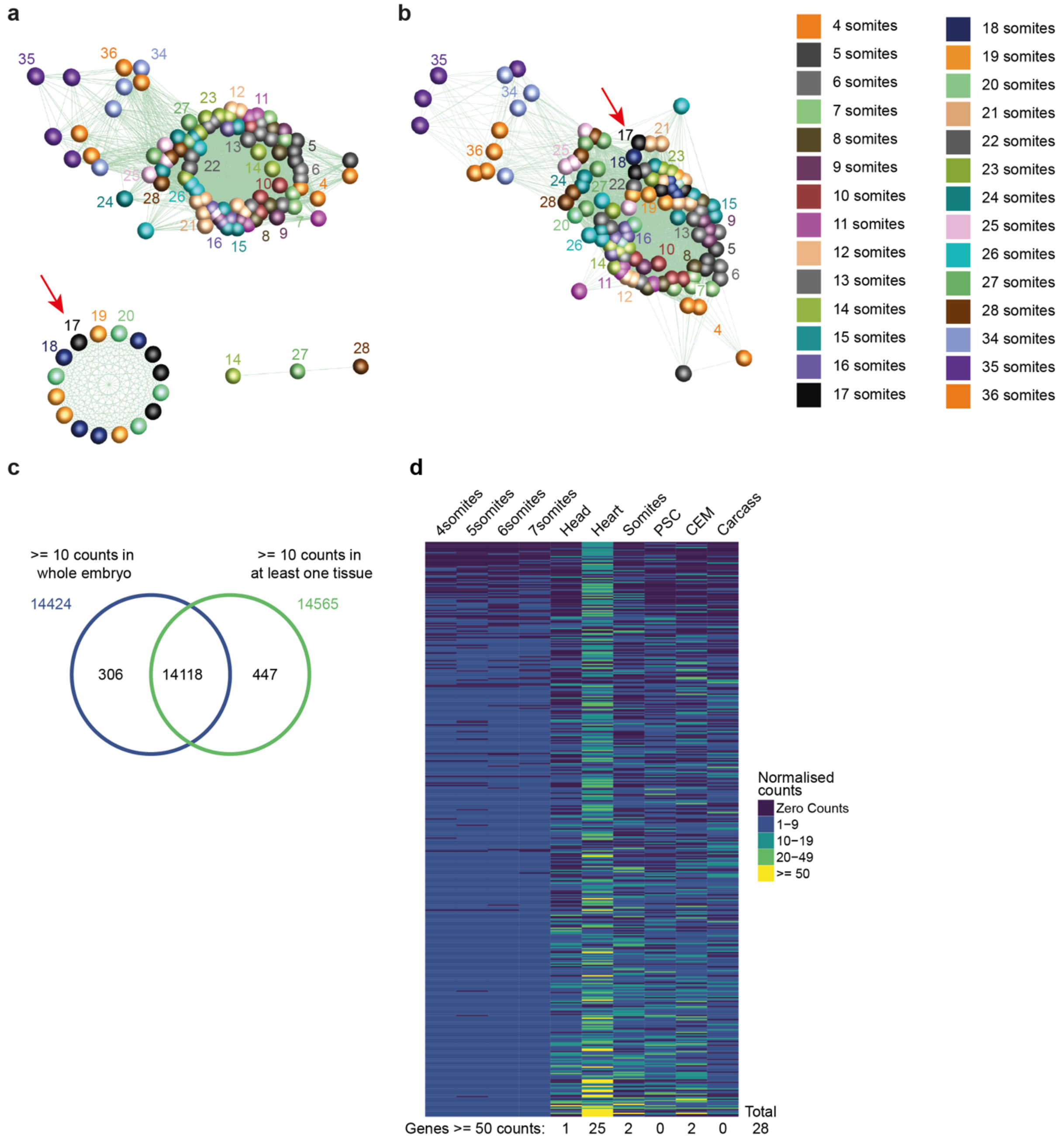

Supplementary Figure 1. Comparison of RNA-seq on dissected tissues and whole embryos.

a–b, Network graphs of baseline samples created by BioLayout Express^{3D}. Each node represents a sample and edges join nodes whose gene expression values over all genes have a Pearson correlation coefficient above a cut-off. The samples are listed in Supplementary Data 1. **a**, Network before removal of batch outlier genes (Pearson cut-off = 0.96). Samples from stages 17-20 somites form a separate network component. **b**, Graph after removal of batch outliers, mitochondrial and sex-specific genes (Pearson cut-off = 0.97). All the samples are now in the same component, for example compare position of samples indicated by red arrows. **c**, Whole embryo sequencing detects the majority of genes. The Venn diagram shows the overlap in the two sets of genes identified in whole embryo and dissected tissue using RNA-seq. **d**, Heatmap of expression levels (normalised counts) of protein-coding genes detected at 10 counts or above in one of the 6 tissues dissected

at E8.25, but at levels below that in all four stage-matched whole-embryo samples (4, 5, 6 and 7 somites). Source data are provided as a Source Data file.

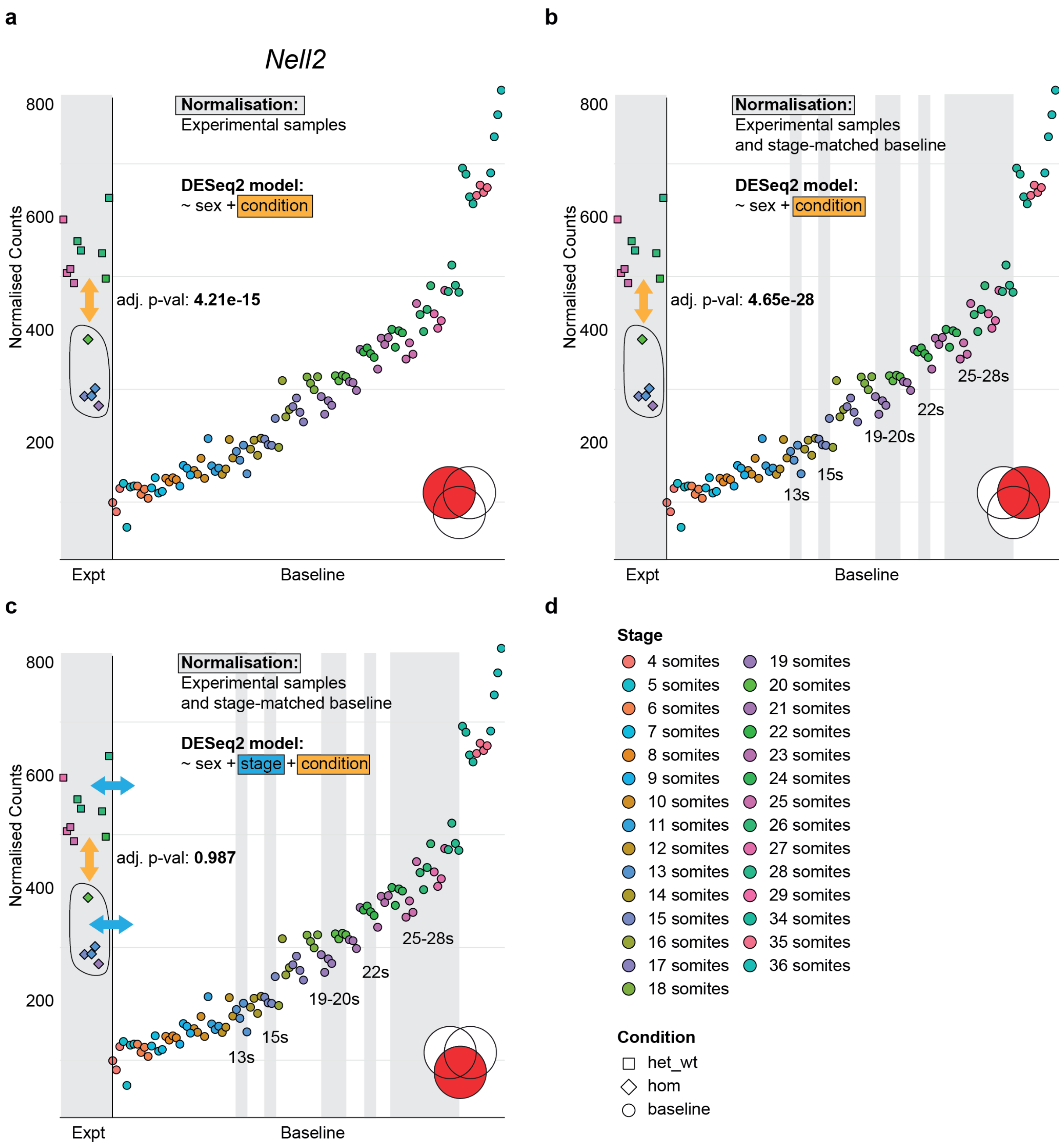

Supplementary Figure 2. Example of DESeq2 delay analysis.

a–c, Plots of normalised counts (for *Nell2*, ENSMUSG00000022454 in the *Brd2* mutant line) depicting how the analysis is run on delayed lines. In each case, the experimental samples (Expt) are shown on the left of the x-axis and all the baseline embryos are on the right. Samples included in a particular run of DESeq2 are shaded in grey. P-values for each run are displayed on each graph. **a**, Only the experimental samples are included (shaded in grey). This compares homozygous embryos to siblings (orange arrow). Genes called as being statistically significant are present in the top left circle of the Venn diagram (red). Corresponds to A in Fig. 2b. **b**, Experimental samples and stage-matched baseline samples (in this case, 13, 15, 19, 20, 22, 25-28 somites) are included (shaded in grey). The same comparison as in **a** is performed, but the inclusion of the baseline samples changes the

dispersion estimate for each gene and thus the calculated p-values. Statistically significant genes are present in the top right circle of the Venn diagram (red). Corresponds to B in Fig. 2b. **c**, Experimental samples and stage-matched baseline samples are included (shaded in grey). In this case, stage is included as a factor in the model (blue arrows). Statistically significant genes are present in the bottom circle of the Venn diagram (red). Corresponds to C in Fig. 2b. In the case of *Nell2*, the difference between homozygous and sibling embryos can be accounted for by stage, so the p-value is greater than 0.05. This gene therefore falls into our “Delay” category (in A and B, but not C). **d**, Legend. Shape; square = sibling, diamond = homozygote, circles = baseline. Source data are provided as a Source Data file.

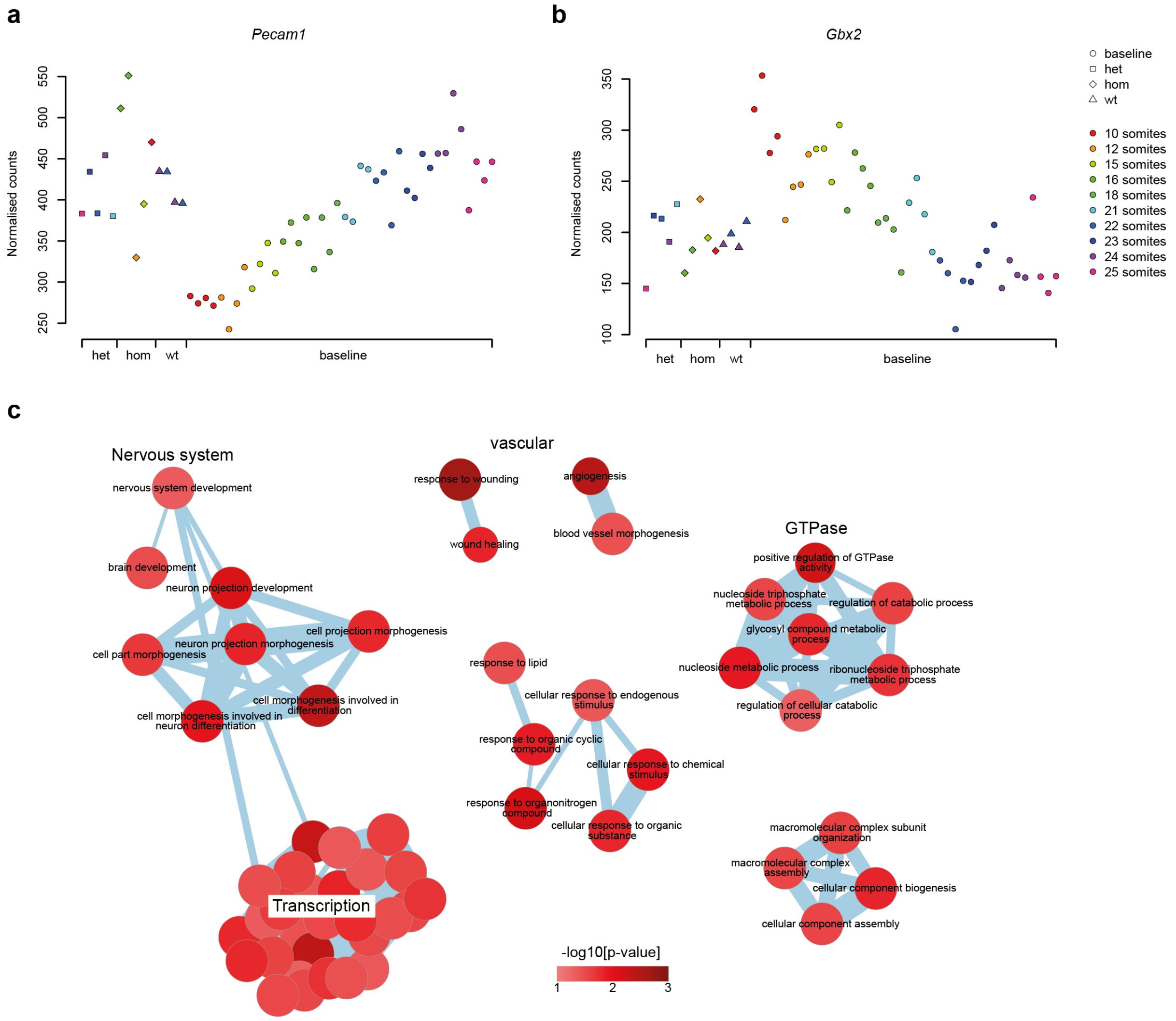

Supplementary Figure 3. No Delay genes.

a–b, Plots of normalised counts showing examples of genes that are expressed at wild-type levels for the appropriate stage. **a**, *Pecam1*. **b**, *Gbx2*. **c**, Network diagram produced by Enrichment Map (Cytoscape App) of the Gene Ontology (GO) term enrichment for genes present in the No Delay list in 6 or more mutant lines. The nodes represent enriched GO terms (Biological Process; fill colour is -$\log_{10}$[pvalue]) and the edge widths are proportional to the overlap of genes annotated to each term. Enriched GO terms that are unconnected to any other enriched terms have been removed. Source data are provided as a Source Data file.

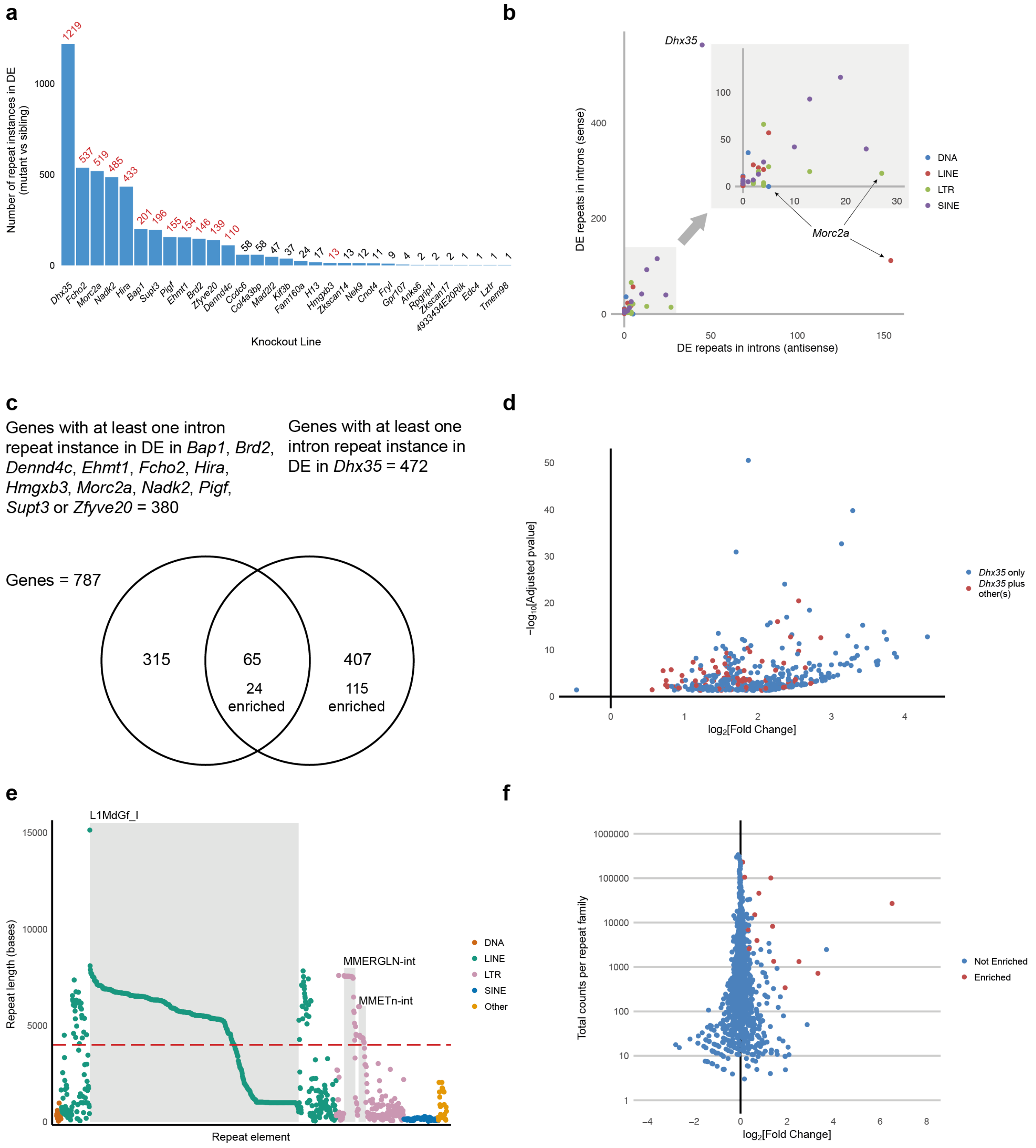

Supplementary Figure 4. Analysis of repeat deregulation in *Dhx35* and *Morc2a* mutant lines.

a, Number of DERs for mutant lines when the reads are mapped to the genome guided by Ensembl transcripts. Above the bars are the number of DERs and red numbers indicates the lines we examined in more detail. **b**, Number of intronic DERs with respect to transcript orientation and repeat group. The number of DERs in the antisense direction is plotted on the x-axis and the number in the sense direction is plotted on the y-axis. The points are coloured by repeat type. **c**, Venn diagram of the numbers of genes containing DERs occurring in introns in the sense orientation across mutant lines. **d**, Volcano plot showing intronic DERs in the sense orientation identified as enriched in genes for the *Dhx35* mutant line. Blue points show DERs unique to *Dhx35*, whereas red points are DERs

found in *Dhx35* and at least one other mutant line. **e**, The genomic span of individual DERs in *Morc2a* mutant with respect to repeat group. Individual DERs are arranged in families on the x-axis and the length in bases of the DER is plotted on the y-axis. The 4000 bp cut-off is displayed as a dashed line. The three repeat families found to be enriched after imposing the ≥4000 bp cut-off are shaded grey and labelled. **f**, Plot showing the overall levels of each repeat family and $\log_2$[fold change] between mutant and wild-type. Each point is a repeat family and those in red are the 14 families where the DERs are enriched (represented in Fig. 5d). Counts for each family were aggregated from individual repeat instances. $\log_2$[fold change] (homozygous versus sibling embryos) is shown on x-axis and total counts for the family are plotted on the y-axis ($\log_{10}$ scaled). Only repeat families where at least one embryo has normalised counts greater than 2 and there are counts in both the homozygous mutant and sibling embryos are shown. Source data are provided as a Source Data file.

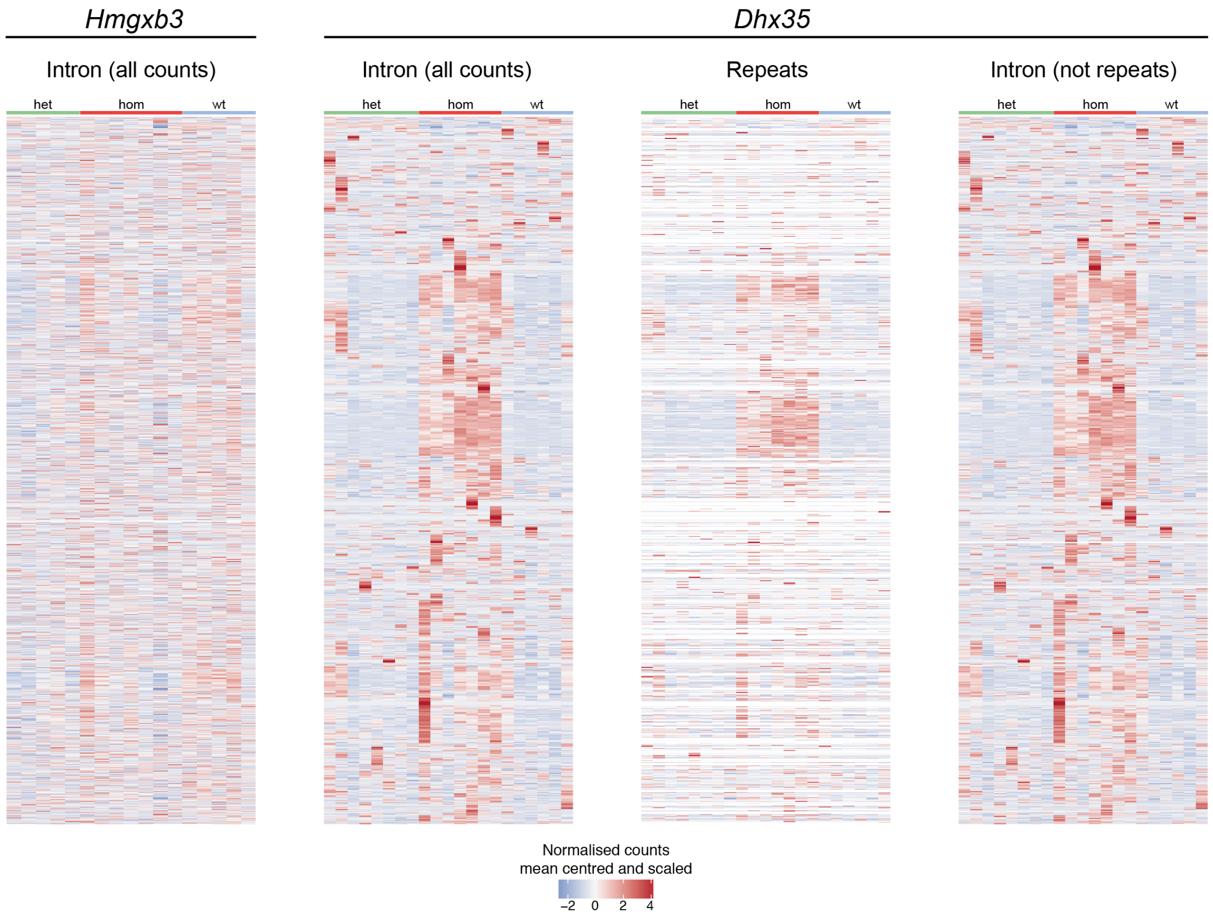

Supplementary Figure 5. Intron retention in *Dhx35*.

All introns from the 115 genes enriched for DERs which are only found in the *Dhx35* mutant line were analysed for counts mapping to introns in three categories; all counts in introns [Intron (all counts)], repeat counts [Repeats] and not repeat counts [Intron (not repeats)], which are the genomic segments between repeats. Counts were normalised using the size factor for each sample and samples which failed QC were removed. *Hmgxb3* was also analysed as a control. All introns with counts in *Dhx35* (rows) are shown in the "Intron (All Counts)" heatmap and are hierarchically clustered using Pearson correlation coefficient as a similarity measure. Introns are in the same order in the other three heatmaps. White cells indicate either zero counts in all samples or introns which don't contain repeats. Samples are grouped by genotype. green = heterozygous (het), red = homozygous (hom) and blue = wild-type (wt) embryos. Source data are provided as a Source Data file.
