# Supplementary material for: Common and distinct transcriptional signatures of mammalian embryonic lethality
Source: Nat Commun. 2019 Jun 26;10:2792. doi: 10.1038/s41467-019-10642-x (PMC6594971; doi:10.1038/s41467-019-10642-x)
Supplement: Supplementary file 9 — Reporting Summary [file 41467_2019_10642_MOESM9_ESM.pdf]

nature research

Corresponding author(s): Elisabeth Busch-Nentwich

Last updated by author(s): Apr 26, 2019

Reporting Summary

Nature Research wishes to improve the reproducibility of the work that we publish. This form provides structure for consistency and transparency in reporting. For further information on Nature Research policies, see Authors & Referees and the Editorial Policy Checklist.

Statistics

For all statistical analyses, confirm that the following items are present in the figure legend, table legend, main text, or Methods section.

n/a	Confirmed	
☐	☒	The exact sample size (*n*) for each experimental group/condition, given as a discrete number and unit of measurement
☐	☒	A statement on whether measurements were taken from distinct samples or whether the same sample was measured repeatedly
☐	☒	The statistical test(s) used AND whether they are one- or two-sided<br>*Only common tests should be described solely by name; describe more complex techniques in the Methods section.*
☒	☐	A description of all covariates tested
☐	☒	A description of any assumptions or corrections, such as tests of normality and adjustment for multiple comparisons
☐	☒	A full description of the statistical parameters including central tendency (e.g. means) or other basic estimates (e.g. regression coefficient) AND variation (e.g. standard deviation) or associated estimates of uncertainty (e.g. confidence intervals)
☐	☒	For null hypothesis testing, the test statistic (e.g. *F*, *t*, *r*) with confidence intervals, effect sizes, degrees of freedom and *P* value noted<br>*Give P values as exact values whenever suitable.*
☒	☐	For Bayesian analysis, information on the choice of priors and Markov chain Monte Carlo settings
☒	☐	For hierarchical and complex designs, identification of the appropriate level for tests and full reporting of outcomes
☐	☒	Estimates of effect sizes (e.g. Cohen's *d*, Pearson's *r*), indicating how they were calculated

Our web collection on statistics for biologists contains articles on many of the points above.

Software and code

Policy information about availability of computer code

Data collection	HiSeq Control Software
Data analysis	TopHat2 v2.0.13<br>QoRTs v1.0.7<br>htseq-count v0.6.0<br>R v3.3.0<br>Cufflinks v2.2.1<br>InterProScan v5.25-64.0<br>iDiffIR https://bitbucket.org/comp_bio/idiffir<br>Ontologizer v2.1<br>Cytoscape v3.5.1<br>custom code for plots https://github.com/richysix/dmdd_figs<br>Baseline CompaRe app https://github.com/richysix/baseline_compare

For manuscripts utilizing custom algorithms or software that are central to the research but not yet described in published literature, software must be made available to editors/reviewers. We strongly encourage code deposition in a community repository (e.g. GitHub). See the Nature Research guidelines for submitting code & software for further information.

Data

Policy information about availability of data

All manuscripts must include a data availability statement. This statement should provide the following information, where applicable:

- Accession codes, unique identifiers, or web links for publicly available datasets
- A list of figures that have associated raw data
- A description of any restrictions on data availability

The authors declare that all data supporting the findings of this study are available within the article and its supplementary information files or from the corresponding author upon reasonable request. All sequencing data have been deposited in ENA under accession codes: ERP010754 [https://www.ebi.ac.uk/ena/data/view/PRJEB9627] (baseline) and ERP008773 [https://www.ebi.ac.uk/ena/data/view/PRJEB7801] (mutant lines). Processed data such as DGE lists and GO/EMAPA enrichments for each mutant line are available at Figshare [10.6084/m9.figshare.c.4127441]. The source data underlying Figs. 1d, 2a,c-e, 3b-d, 4a-c, 5c, 6b-d, and Supplementary Figs. 1d, 2, 3, 4a-b, 5a-b,d-f, 6 are provided as a Source Data File.

Field-specific reporting

Please select the one below that is the best fit for your research. If you are not sure, read the appropriate sections before making your selection.

☒ Life sciences ☐ Behavioural & social sciences ☐ Ecological, evolutionary & environmental sciences

For a reference copy of the document with all sections, see nature.com/documents/nr-reporting-summary-flat.pdf

Life sciences study design

All studies must disclose on these points even when the disclosure is negative.

Sample size	Sample sizes were not pre-determined and were dependent on the mouse knock-out production pipeline.
Data exclusions	Samples were excluded based on the quality of each sequencing library which was manually assessed one knock-out line at a time using the output of QoRTs (measures used were insert size, GC content and proper pairs).
Replication	No replications were performed due to the high-throughput nature of the study.
Randomization	No randomization was required in the study.
Blinding	No blinding was required in the study.

Reporting for specific materials, systems and methods

We require information from authors about some types of materials, experimental systems and methods used in many studies. Here, indicate whether each material, system or method listed is relevant to your study. If you are not sure if a list item applies to your research, read the appropriate section before selecting a response.

Materials & experimental systems

n/a	Involved in the study	
☒	☐	Antibodies
☒	☐	Eukaryotic cell lines
☒	☐	Palaeontology
☐	☒	Animals and other organisms
☒	☐	Human research participants
☒	☐	Clinical data

Methods

n/a	Involved in the study	
☒	☐	ChIP-seq
☒	☐	Flow cytometry
☒	☐	MRI-based neuroimaging

Animals and other organisms

Policy information about studies involving animals; ARRIVE guidelines recommended for reporting animal research

Laboratory animals	Mus musculus, C57BL/6N, E9.5
Wild animals	The study did not involve wild animals
Field-collected samples	The study did not involve samples collected form the field
Ethics oversight	Wellcome Sanger Institute's Animal Welfare and Ethical Review Body

Note that full information on the approval of the study protocol must also be provided in the manuscript.
