# Supplementary material for: Common and distinct transcriptional signatures of mammalian embryonic lethality
Source: Nat Commun. 2019 Jun 26;10:2792. doi: 10.1038/s41467-019-10642-x (PMC6594971; doi:10.1038/s41467-019-10642-x)
Supplement: Supplementary file 11 — Description of Additional Supplementary Files [file 41467_2019_10642_MOESM11_ESM.pdf]

Supplementary Data Legends

Supplementary Data 1. Baseline sample information.

Sample Name: sample name.

Sample Accession: European Nucleotide Archive sample accession.

Number of Somites: recorded number of somites of the embryo.

Sex: sex of the embryo (F/M).

QC Outcome: outcome of QoRTS QC (pass/fail).

Reads mapped: Number of reads mapped to GRCm38.

Estimated Library Size: Estimate of library size from biobambam bammarkduplicates

Supplementary Data 2. Genes removed as technical batch effect.

Ensembl Gene ID: Ensembl id of the gene.

Chr: Chromosome.

Start: Start of gene on chromosome. Always the smaller of the two values irrespective of the direction of transcription of the gene.

End: End of gene.

Strand: Direction of transcription. 1 = forward strand, -1 = reverse strand.

Biotype: Gene biotype (https://www.ensembl.org/info/genome/genebuild/biotypes.html)

Name: Gene name.

Description: Gene description.

Supplementary Data 3. Novel genes.

GFF3 format (see https://www.ensembl.org/info/website/upload/gff3.html)

seqid: name of the chromosome or scaffold.

source: name of the program that generated this feature

type: type of feature.

start: Start position of the feature (1-based).

end: End position of the feature (1-based).

score: No scores so all values are "."

strand: defined as + (forward) or: - (reverse).

phase: No phase data so all values are "."

attributes: A semicolon-separated list of tag-value pairs, providing additional information about each feature. tags are

> ID: an id for the transcript
>
> geneID: an id for the gene
>
> oId: id use by cufflinks
>
> class_code: a code used by cufflinks for the type of transcript.
> In this case they are all "u" for "Unknown, intergenic transcript"
>
> tss_id: The ID of this transcript's inferred start site

biotype: type of transcript e.g. protein_coding

Supplementary Data 4. Sample information for the mutant lines.

Mutant Gene: Name of the targeted gene.

Ensembl ID: Ensembl gene id of the targeted gene.

Allele: Allele designation.

Number of Somites: recorded number of somites of the embryo.

Sex: sex of the embryo (F/M).

Genotype: genotype of the embryo. (hom/het/wt)

Sample Name: sample name.

Sample Accession: European Nucleotide Archive sample accession.

QC Outcome: outcome of QoRTS QC (pass/fail).

Reads mapped: Number of reads mapped to GRCm38.

Estimated Library Size: Estimate of library size from biobambam bammarkduplicates

Supplementary Data 5. Genes that contribute 50% of the variation explained by PC3.

Gene ID: Ensembl gene id

% contribution: Contribution of the expression of this gene to PC3 as a percentage. Cumulatively, these genes contribute 50% of the variance explained by PC3.

Name: Gene name.

Description: Gene description.

Chr: Chromosome.

Start: Start of gene on chromosome. Always the smaller of the two values irrespective of the direction of transcription of the gene.

End: End of gene.

Strand: Direction of transcription. 1 = forward strand, -1 = reverse strand.

Biotype: Gene biotype (https://www.ensembl.org/info/genome/genebuild/biotypes.html)

Supplementary Data 6. Genes frequently present in the No Delay category.

Sheet1: gene_list. The 196 genes that are present in the No Delay category in more than six lines.

Gene stable ID: Ensembl id of the gene.

Gene name: Name of the gene

Gene description: Description of the gene.

Sheet2: Gene Ontology (GO) term enrichment of the gene list.

GO.ID: Id of the GO term.

Term: Description of the term.

Annotated: The number of genes annotated to the GO term.

Significant: The number of genes annotated to the GO term that appear in the gene list.

Expected: The number of genes annotated to the GO term that would be expected to appear in the gene list by chance given the size of the gene list.

pval: enrichment test p-value.

Supplementary Data 7. Expression of L1MdGf_I repeats in baseline embryos.

Name: Name of the repeat instance.

Chr: Chromosome.

Start: Start of gene on chromosome. Always the smaller of the two values irrespective of the direction of transcription of the gene.

End: End of gene.

Strand: Direction of transcription. 1 = forward strand, -1 = reverse strand.

Class: Repeat class. These are all LINE/L1s.

The remaining columns contain the raw counts and DESeq2 normalised counts for the baseline samples. Columns are named “<sample name> count” and “<sample name> normalised count”

mean normalised counts: The last column is the mean of all the normalised counts.
